# Detection of Lymphatic Vessels in the Superficial Fascia of the Abdomen

**DOI:** 10.3390/life13030836

**Published:** 2023-03-20

**Authors:** Giovanna Albertin, Laura Astolfi, Caterina Fede, Edi Simoni, Martina Contran, Lucia Petrelli, Cesare Tiengo, Diego Guidolin, Raffaele De Caro, Carla Stecco

**Affiliations:** 1 Department of Neuroscience (DNS), Section of Human Anatomy, University of Padova, 35122 Padova, Italy; 2 Bioacoustics Research Laboratory, Department of Neuroscience (DNS), University of Padova, 35129 Padova, Italy; 3 Interdepartmental Research Center of International Auditory Processing Project in Venice (I-APPROVE), Department of Neurosciences, University of Padova, Santi Giovanni e Paolo Hospital, ULSS3 Serenissima, 30122 Venezia, Italy; 4 Clinic of Plastic Surgery, Padova University Hospital, 35128 Padova, Italy

**Keywords:** lymphatics vessels, subcutaneous tissue, reconstructive surgery, superficial fascia, hypodermis, immunohistochemistry, lymphatic system

## Abstract

Recently, the superficial fascia has been recognized as a specific anatomical structure between the two adipose layers—the superficial adipose tissue (SAT) and the deep adipose tissue (DAT). The evaluation of specific characteristics of cells, fibers, blood circulation, and innervation has shown that the superficial fascia has a clear and distinct anatomical identity, but knowledge about lymphatic vessels in relation to the superficial fascia has not been described. The aim of this study was to evaluate the presence of lymphatic vessels in the hypodermis, with a specific focus on the superficial fascia and in relation to the layered subdivision of the subcutaneous tissue into SAT and DAT. Tissue specimens were harvested from three adult volunteer patients during abdominoplasty and stained with D2-40 antibody for the lymphatic endothelium. In the papillary dermis, a huge presence of lymphatic vessels was highlighted, parallel to the skin surface and embedded in the loose connective tissue. In the superficial adipose tissue, thin lymphatic vessels (mean diameter of 11.6 ± 7.71 µm) were found, close to the fibrous septa connecting the dermis to the deeper layers. The deep adipose tissue showed a comparable overall content of lymphatic vessels with respect to the superficial layer; they followed the blood vessel and had a larger diameter. In the superficial fascia, the lymphatic vessels showed higher density and a larger diameter, in both the longitudinal and transverse directions along the fibers, as well as vessels that intertwined with one another, forming a rich network of vessels. This study demonstrated a different distribution of the lymphatic vessels in the various subcutaneous layers, especially in the superficial fascia, and the demonstration of the variable gauge of the vessels leads us to believe that they play different functional roles in the collection and transport of interstitial fluid—important factors in various surgical and rehabilitation fields.

## 1. Introduction

The lymphatic system is distributed throughout the entire human body, and the superficial lymphatic circulation consists of dermal lymphatic capillaries and subcutaneous lymph-collecting vessels. The main role of the lymphatic system is to return protein deposits and extra tissue fluid extravasated from the blood capillaries to the interstitial tissues, feeding into the blood circulation system to maintain fluid balance in the body. The initial lymphatic vessels (LVs) that form in the dermis are composed of a thin layer of endothelial cells that are physically tethered to the surrounding extracellular matrix, along with gradual collection vessels adapted to ensure transport of lymph formed in deep tissues [1]. Lymphedema [2,3,4] is a clinical manifestation caused by impaired lymphatic transport or by the chronic accumulation of interstitial fluid that leads to adipose deposition, fibrosis, or persistent inflammation in the subcutis [2]. Today, therapies for lymphedema—such as liposuction or lymph node transplantation—require a detailed understanding of the anatomy of the subcutaneous lymphatic system. The localization of the LVs and their organization in the subcutaneous layers can help in understanding their altered morphological development in this pathology, as the basis of the fluid accumulation process in interstitial spaces [3]. At the same time, the analysis of the LVs’ distribution can be useful to improve surgical procedures that can preserve the distribution of lymphatic vessels [4].

In the past, the hypodermis was considered to be a homogeneous structure filling the space between the skin and the muscles, but recently many studies have highlighted that it is divided into two layers—the superficial adipose tissue (SAT) and deep adipose tissue (DAT) [5,6,7]—by the superficial fascia (SF). The superficial fascia, recognized as a specific anatomical structure with a distinct anatomical identity, presents specific characteristics of cells and innervation; it is a continuous thin fibrous membrane that is rich in elastic fibers, with a mean thickness of 847.4 ± 295 μm [8,9]. The SAT [8] is a well-organized adipose layer, organized in polygonal–oval fat lobules with vertical fibrous septa, whilst in the DAT the fibrous septa are mostly obliquely/horizontally oriented and the fat cells are few and less organized. The DAT is lipolytically more active [10], with a higher ratio of saturated to monounsaturated fatty acids compared to the SAT [11]. Its function is to provide the autonomy of the SF with respect to the deep fascia, creating a gliding surface between the subcutaneous layer and the musculoskeletal system [12]. Cancello et al. [13] have shown that, in obese patients, the abdominal SAT and DAT subcompartments are different at both the molecular and morphological levels, with significant adipocyte hypertrophy in the SAT compared to the DAT. Adiponectin is preferentially expressed in the SAT, whereas inflammatory genes are overexpressed in the DAT. Consequently, the subcompartments have to be considered independently when investigating the subcutis biology and clinical complications of obesity [13].

Today, the exact localization of the LVs in the SAT and DAT, along with their relationships with the superficial fascia, is not well defined. Kubik and Manestar [14] reported that the LVs of the thigh formed three layers: a first layer immediately below the surface of the subcutaneous fat, a second layer between the first and third layers, and a third layer in the deep fascia; however, they did not clarify exactly where the second layer is located. According to Tourani et al. [15], in the abdomen, the collectors were found above Scarpa’s fascia immediately below the subdermal venules. They were thin-walled and translucent, and their diameter ranged between 0.2 and 0.8 mm. In the upper thigh, the same authors found two distinct groups of superficial collectors: one deeper, with thick walls measuring 0.6–1 mm in diameter; and one superficial, immediately below the subdermal venules, with thin walls measuring 0.3–0.5 mm. Moreover, Culligan et al. [16] demonstrated that small LVs exist within Toldt’s fascia, which separates all apposed portions of the mesocolon from the underlying retroperitoneum. They found a lymphatic network within the connective tissue of the mesenteric organs, and this specific knowledge is important for the decision as to whether the fascial tissues should be removed in oncological colorectal resections. Onder et al. [17] highlighted the interaction between mesenchymal cells and lymphatic endothelial cells in the space close to the deep fascia, suggesting that it could be crucial for the activation of lymph node development [17]. Furthermore, Hayashida et al. [18] evaluated a combined treatment of adipose-derived stem cells and vascularized lymph node transfer for decreasing edema volumes through accelerated lymphatic drainage.

This layered organization of the subcutaneous tissue has recently attracted increasing interest in surgical procedures, such as flaps in plastic reconstructive surgery and fat removal techniques in aesthetic surgery (e.g., abdominoplasty procedures and liposuction). Recently, a new surgical technique for abdominoplasty has been developed, focused on the preservation of Scarpa’s fascia and the deep fat compartment, along with their lymphatic and blood vessels [19,20,21]. This enables a reduction in the incidence of seroma—the most frequent complication related to surgery in the lower abdomen [21].

The aim and the novelty of this research is to understand and describe the lymphatic vessels’ distribution, density, diameter, and organization layer by layer, from the dermis to the DAT, with a particular focus on the superficial fascia.

## 2. Materials and Methods

### 2.1. Sample Collection

Tissue specimens were harvested from three adult volunteer patients (mean age 42 ± 4 years; 2 females, 1 male) during abdominoplastic surgery. All of the patients were obese (BMI 28–30), without any comorbidities reported (such as diabetes, cardiovascular disease, or cancer). The ethical regulations regarding research on human tissues were carefully followed in accordance with the rules described by Macchi and co-authors [22]. According to Italian law, the parts of the body removed for therapeutic purposes during surgery that would otherwise be destined for destruction can be used for research. For each patient, 3 samples, of ~1 cm^2^ were randomly collected from different areas of the abdominal region, at full thickness, from the epidermis to the DAT [23]. Moreover, from each subject, three additional samples of the superficial fascia were isolated from the surrounding adipose tissue. Next, the samples were fixed in 10% buffered formaldehyde (pH 7.4), after which they were dehydrated in graded ethanol and xylene, and then embedded in paraffin. Five-micrometer sections were cut using a microtome for histological and immunohistochemical staining.

### 2.2. Histological and Immunohistochemical Staining

First, the sections were stained with hematoxylin and eosin to evaluate the general morphology of the tissue. Secondly, the sections were additionally immunostained with the monoclonal antibody D2-40 (Biocare Medical, Cat.# CM 266 A, B, C, RRID: AB_2923154) against the transmembrane glycoprotein podoplanin, as a specific marker for the endothelial cells of the LV [24,25,26], according to the following protocol. Dewaxed sections were treated with 1% hydrogen peroxide (Marco Viti Pharmaceutical, Sandrigo, Vicenza, Italy) to block endogenous peroxidases. Non-specific sites were then saturated with horse serum (S-2000, Vector Laboratories, Burlingame, CA, USA) (HS) and diluted 1:60 in triphosphate buffer solution + 0.1% Tween 20 (Sigma-Aldrich, St. Louis, MI, USA) (TBST) for 1 h. Then, the sections were incubated with the murine monoclonal primary antibody D2-40 and diluted 1:100 in TBST + 1% HS overnight at 4 °C. After repeated washing with TBST, the sections were incubated with a horse anti-mouse secondary antibody (BA-2000, Vector Laboratories) and diluted 1:500 in TBST for 30 min. Signal amplification was performed using the ABC-HRP Kit, Peroxidase (Standard) (PK-6100, Vector Laboratories), and the reaction was developed with 3,3′-diaminobenzidine (Liquid DAB + substrate Chromogen System (K346711-2, Agilent’s Dako, Santa Clara, CA, USA)) before being stopped with distilled water. Nuclei were counterstained with Mayer’s hematoxylin (Leica Microsystems, Milan, Italy). Skin samples on which the primary antibody was omitted were used as negative controls, and human tonsil samples were used as positive controls, to verify the specificity of the reaction.

### 2.3. Image Acquisition

The images were acquired using Leica DMR microscope (Leica Microsystems, Wetzlar, Germany) operating at a primary magnification of 10×. To ensure systematic scanning of the tissue, one operator counted all of the fields, starting from the upper-left corner and moving horizontally from left to right, then one row down from right to left, and so on. The total number of acquired images was recorded, and the D2-40-positive fields were counted with respect to the total section and with respect to the various subcutaneous layers (D: epidermis and dermis; SAT: superficial adipose tissue; SF: superficial fascia; DAT: deep adipose tissue). A second image acquisition at 20× magnification was performed to acquire images on each positive field, with the aim of measuring the diameters of the vessels. The images were acquired in full color (RGB, 24-bit) and saved as TIFF files for further processing.

### 2.4. Image Analysis of Lymphatic Vessels

Computer-assisted image analysis was performed on the obtained set of images to characterize D2-40 immunoreactivity in the subcutaneous layers of full-thickness samples. All analysis procedures were performed using ImageJ software, freely available at http://rsb.info.nih.gov/ij/ (accessed on 16 March 2023) [27]. Briefly, after shading correction and contrast enhancement, color thresholding was applied to discriminate immunoreactive structures, and the area occupied by immunoreactive structures in each layer (D, SAT, SF, and DAT) was then measured. From these primary data, two parameters were derived: The first describes the percentage of the tissue area in a layer occupied by immunoreactivity (Area%), which was estimated from the ratio between the total immunoreactive area measured in that layer and the total sampled area of the layer. The second provides information on the relative amount of immunoreactive structures in each layer (IR%), which was evaluated as the percentage ratio between the total immunoreactive area measured in that layer and the total immunoreactive area observed in the whole section. Finally, in each of the considered tissue layers, the mean diameter of D2-40-positive vessels was evaluated by interactively measuring this parameter on a sample (*n* = 30) of immunoreactive profiles.

### 2.5. Statistics

For each layer, the obtained Area%, IR%, and diameter values were averaged across the 9 samples analyzed to provide representative values of the parameters for that layer. With the sample size being quite small, the normality of the datasets was first verified using the Kolmogorov–Smirnoff method. Differences between the four considered layers were then statistically tested by one-way analysis of variance, followed by Tukey’s test for multiple comparisons. The GraphPad Prism 3.0 statistical package (GraphPad Software Inc., San Diego CA, USA) was used for the analysis. Results are reported as the mean ± SEM, and *p* < 0.05 was considered as the limit for statistical significance.

## 3. Results

### 3.1. Morphological Structure of the Samples

**Figure 1:**
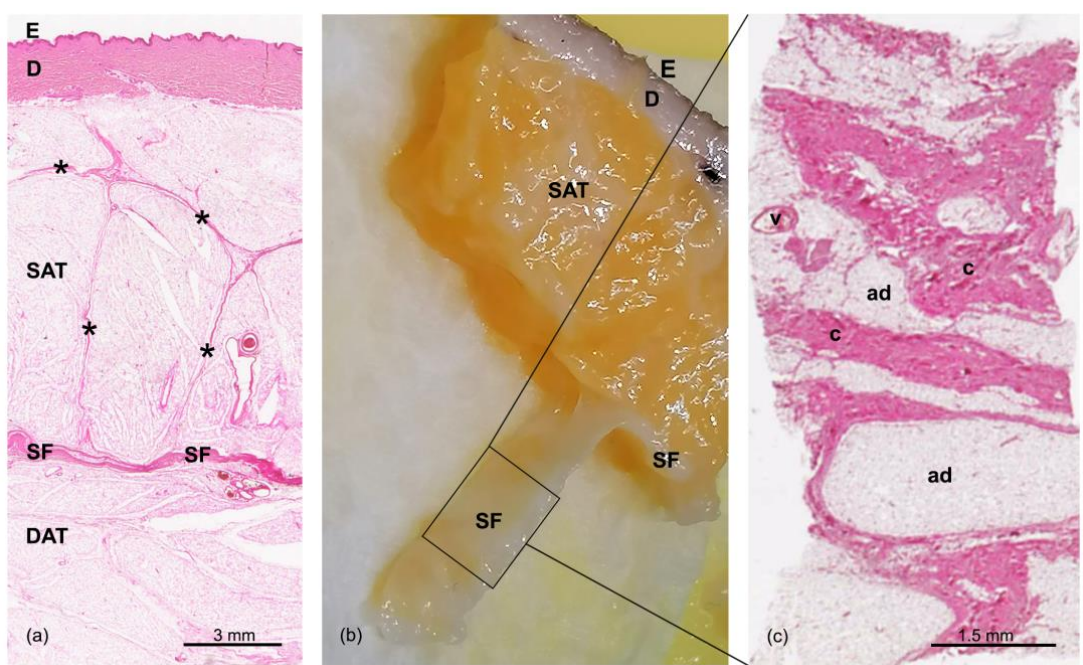
Skin and the subcutaneous layers of the abdomen: (**a**) Hematoxylin and eosin staining of a full-thickness specimen, in which we can see the epidermis (E) and dermis (D); the superficial adipose tissue (SAT), organized in lobules separated by the fibrous septa of the retinacula cutis (asterisks); the superficial fascia (SF); and the deep adipose tissue (DAT). (**b**) Formalin-fixed sample of the abdominal region (E: epidermis, D: dermis; SAT: superficial adipose tissue, SF: superficial fascia). The SF—the layer localized between the SAT and DAT—was isolated and stained with hematoxylin and eosin. (**c**) A tangential section of the flat embedded SF shown in panel (**b**). The SF is formed by a network of collagen fibers arranged irregularly (c: connective tissue), interconnected and mixed with adipocytes (ad), and crossed by blood vessels (v). Scale bars: (**a**) = 3 mm; (**b**) = 1.5 mm.

The subcutaneous layers were clearly recognizable in all of the samples stained with hematoxylin and eosin (Figure 1). More specifically, from the surface going inward, the following layers could be identified: the skin (epidermis (E) and dermis (D)), the superficial adipose tissue (SAT), the superficial fascia (SF), and the deep adipose tissue (DAT). There were no identified differences between the specimens derived from different subjects.

The staining of the dermis highlights the fibrillary component that then continues as fibrous septa (retinacula cutis superficialis) into the SAT. These septa appeared well defined, with a perpendicular orientation with respect to the surface; they encased the subcutaneous fat in large adipose lobes, as a honeycomb structure (Figure 1a). Below, the SF appeared as a thin fibrous layer (Figure 1b), with a membranous appearance and a multilayered structure of collagen bundles. The histological tangential section of the SF layer showed a net of irregularly arranged collagen fibers, interpenetrated by adipose clusters and crossed by a rich vascular pattern (Figure 1c), as already demonstrated and described in our previous works [23,28]. Beneath the SF, another layer of adipose tissue was visible—the DAT—with smaller, flatter, and less-defined adipose lobes than those of the SAT (Figure 1a). Furthermore, the network of collagen fibrous septa (retinacula cutis profunda) that was visible in this layer was more irregular.

### 3.2. Distribution of Lymphatic Vessels

**Figure 2:**
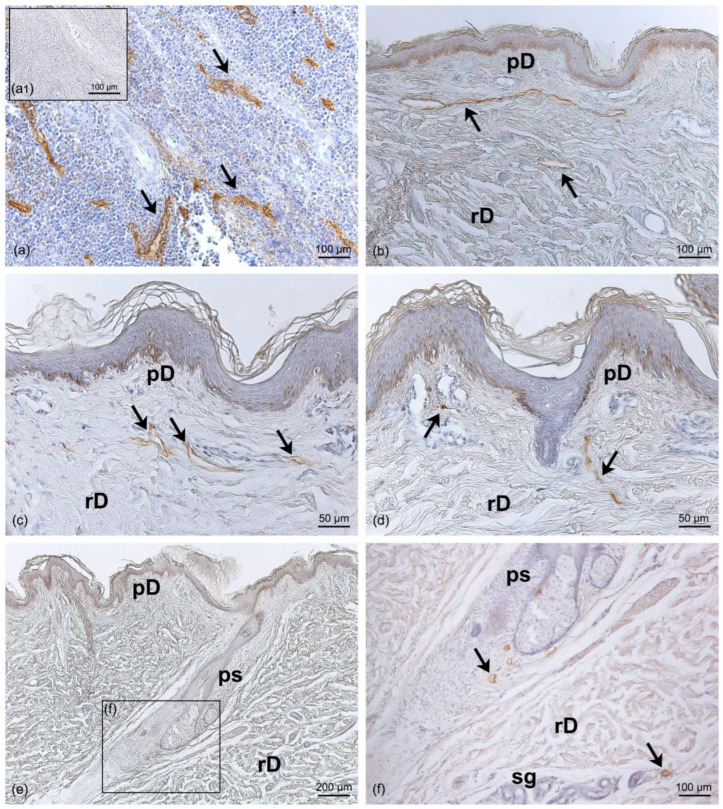
Immunohistochemistry with D2-40 antibody on full-thickness specimens of the dermis of the abdominal region: (**a**) Positive control of immunoreaction for the D2-40 LV marker in human tonsil tissue, where the brown staining (arrows) highlights the LVs in the endothelium between the lymphoid tissues; (**a1**) inset of negative control with human tonsil tissue by omission of the D2-40 antibody. (**b**–**d**) The distribution of LVs follows different orientations in the papillary dermis (pD) and the reticular dermis (rD) (arrows). (**e**) Full length of the pilosebaceous structure (ps) from the epidermis to the reticular dermis; the magnification in (**f**) shows LVs close to the pilosebaceous structure and sweat glands (sg) (black arrow). Scale bars: (**a**,**b**,**a1**,**f**) = 100 µm; (**c**,**d**) = 50 µm; (**e**) = 200 µm.

**Figure 3:**
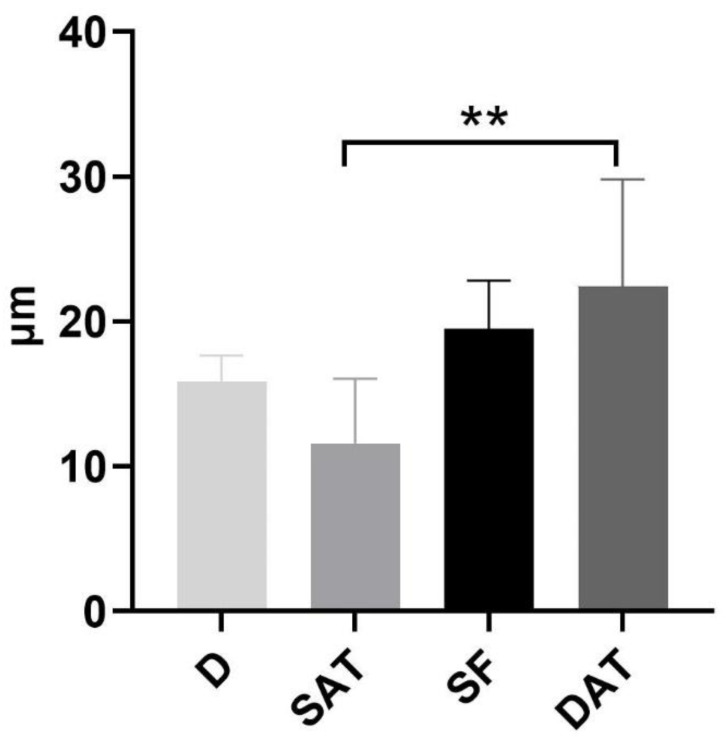
Mean diameter (µm) of LVs in specific areas—D, SAT, SF, and DAT. ** = Multiple comparison analyses found a significant difference in the vessel diameters between the SAT and the DAT (*p* = 0.038).

The positivity of the LV endothelium marker D2-40 enabled us to distinguish the LVs from the blood vessels. In the LVs, the endothelium appeared positive for the staining and was morphologically extremely flattened, with an empty lumen (Figure 2). The specificity of the staining was tested in a human tonsil tissue specimen, used as a positive control (Figure 2a). The D2-40 antibody strongly marked the vessels observed in the papillary dermis and embedded in the loose connective tissue (Figure 2b–d). LVs crossed the tissue vertically to reach the deeper dermis (Figure 2d), where LVs were also observed near the sebaceous glands, the hair follicles, and the sweat glands, with a coiled tubular structure (Figure 2e,f). The evaluation of the diameter of LVs in the dermis showed a mean value of 15.5 ± 3.42 µm (Figure 3).

**Figure 4:**
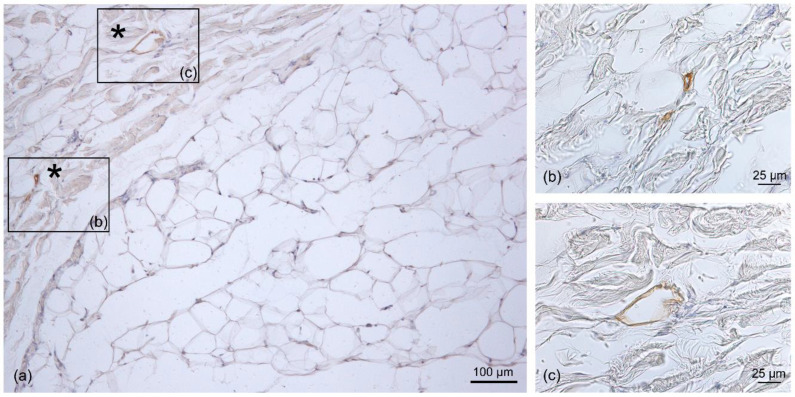
Immunohistochemistry with D2-40 antibody on a full-thickness-specimen of the SAT layer: (**a**) immunostaining of lymphatic vessels between collagen fibers (asterisk) at the fibrous septa level; (**b**,**c**) 40× magnification of the vessels. Scale bars: (**a**) = 100 µm; (**b**,**c**) = 25 µm.

In the SAT, only thin LVs were visible; they were found close to the fibrous septa (retinacula cutis) connecting the dermis and the SAT, as well as around the blood vessels (Figure 4). The mean diameter of the LVs in the SAT area was equal to 11.6 ± 7.71 µm (Figure 3).

**Figure 5:**
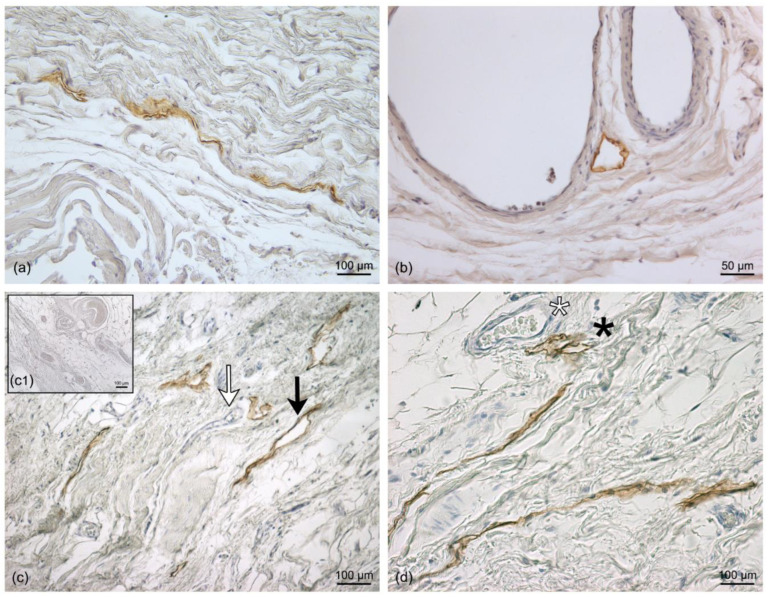
Immunohistochemistry with D2-40 antibody on the superficial fascia (SF) layer: The LVs follow the collagen fibers (**a**) and are distributed close to the blood vessels (**b**). (**c**,**d**) Several sections of LVs (black arrow) and blood vessels (white arrows) between the collagen fibers (**c**) of the SF and the transverse section of the LVs (black asterisk) close to the transverse section of the blood vessels (white asterisk) (**d**); (**c1**) inset of the negative control with SF by omission of the D2-40 antibody. Scale bars: (**a**,**c**,**c1**,**d**) = 100 µm; (**b**) = 50 µm.

In the superficial fascia layer, more LVs were evident, and with different orientations; they followed the fibers, and they were distributed in both the longitudinal and transverse directions (Figure 5). The mean diameter of these intrafascial LVs was 19.5 ± 5.77 µm (Figure 3).

**Figure 6:**
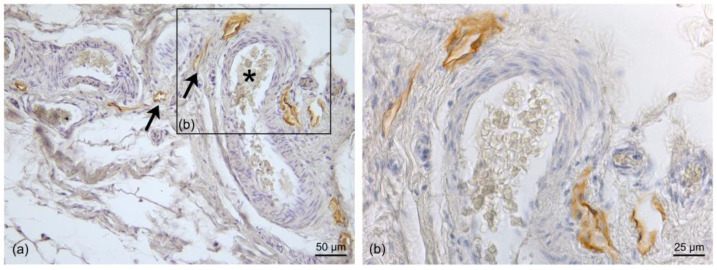
Immunohistochemistry with D2-40 antibody in a full-thickness specimen f the DAT layer: (**a**) Positive immunostaining of LVs (black arrow) close to the blood vessels (black asterisk); in the blood vessels, the endothelium is not stained, and large amounts of erythrocytes are visible in the lumen; (**b**) 20× Magnification: blood vessels with open LVs around them. Scale bars: (**a**) = 50 µm; (**b**) = 25 µm.

Lastly, the DAT presented clear and wide LVs close to the blood vessels (Figure 6). These LVs were less connected with the fibrous septa, and the mean diameter of the vessels in this region was 22.5 ± 12.77 µm (Figure 3).

Multiple comparison analyses showed a significant difference in the vessel diameters between the SAT and the DAT (*p* = 0.038), and both the SAT and DAT diameters showed no significant difference with the SF (Figure 3).

**Figure 7:**
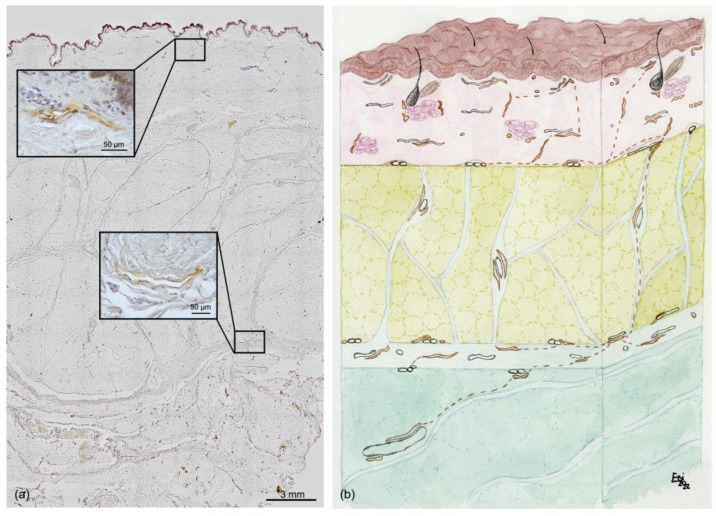
Immunohistochemistry and schematic drawing of the distribution of LVs in the subcutis: (**a**) Immunohistochemistry with D2-40 antibody on a full-thickness, with two high-magnification examples of staining with D2-40 (in the insets), on the dermis and superficial fascia, respectively (scale bar: 50 µm). (**b**) Schematic drawing of the distribution of LVs (rust color) in the skin and subcutaneous tissue. The LVs’ organization follows the layer organization. A first LV plexus is present at the dermis level up to the dermis border. Our results may indicate the presence of LVs with a vertical course following the retinacula cutis (dotted line) in the SAT; the vessels connect to form a second plexus in the superficial band and, even deeper, in the DAT, where the vessels go to form LVs of greater caliber (i.e., collectors) (scale bar: 200 µm).

Figure 7a shows a full-thickness sample from the skin to the DAT, stained with D2-40; the immunoreactivity was visible only at greater magnification (as shown in the insets). Figure 7b summarizes and illustrates the organization of the LVs in relation to the superficial fascia.

### 3.3. Density Analysis of Lymphatic Vessels

**Figure 8:**
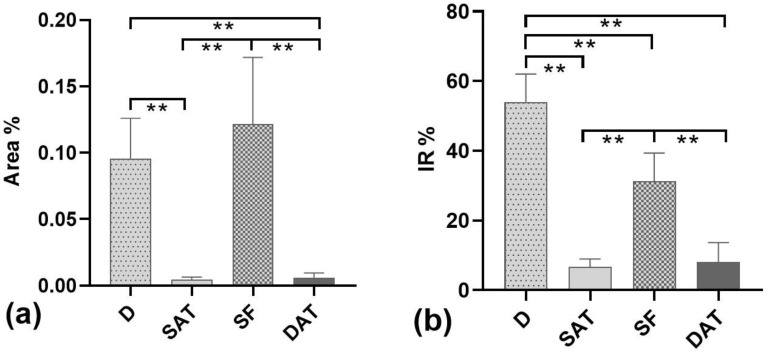
Immunoreactivity % in the cutis and subcutis. (**a**) Percentage of the tissue area covered by immunoreactivity (Area%). (**b**) Percentages of immunoreactive structures (IR%) in the dermis (D), superficial adipose tissue (SAT), superficial fascia (SF), and deep adipose tissue (DAT); ** = *p* < 0.05 according to multiple comparison analyses.

The density of the LVs was analyzed in each layer. The Area% shown in Figure 8a was estimated from the ratio between the total immunoreactive area measured in a specific layer and the total sampled area of the same layer. The results showed that the Area% in the dermis (D) (0.095 ± 0.018%) was significantly higher than in the SAT and DAT (*p* < 0.01), but it was not significantly different with respect to the SF layer (0.122 ± 0.029%; *p* > 0.05) (Figure 8a). The density of LVs in the DAT was not significantly different (*p* > 0.05) than in the SAT, with a mean Area% of 0.0059 ± 0.002% (from 0.0018 to 0.013) and 0.0044 ± 0.001% (from 0.0023 to 0.0084), respectively (Figure 8a).

The SF layer was the second most reactive tissue, after the dermis, when evaluating the percentage ratio of LV positivity with respect to the total immunoreactive area observed in the whole section (*p* = 0.0008 between D and SF) (IR%, Figure 8b). In fact, the IR% was equal to 54.03 ± 13.8% in the dermis, 31.2 ± 14.1% in the SF, 6.67% ± 3.9 in the SAT, and 8.07% ± 9.6 in the DAT.

## 4. Discussion

This study demonstrated for the first time the presence of lymphatic vessels inside the superficial fascia, and that the LVs have a different distribution in the various subcutaneous layers (Figure 8).

We confirmed the presence of a lymphatic plexus extended into the dermal papillae, as already described by Ryan [29], but this study also highlighted for the first time a second plexus inside the superficial fascia. In the SF, a huge number of LVs (IR% equal to 31.2 ± 14.1%, with a mean diameter of 19.5 ± 5.77 µm) were clearly identifiable, with different spatial orientations, supporting the idea that they are organized in a sort of lymphatic plexus that then feeds into the LVs of the DAT. This description of a new lymphatic plexus inside the SF could explain why the preservation of the Scarpa and DAT layers during abdominal surgery can reduce the incidence of seroma [21].

Furthermore, the LVs in the SAT and DAT also showed peculiar characteristics and specific relationships with the surrounding structures. Indeed, in the SAT, the LVs had a vertical course and followed the retinacula cutis, whilst in the DAT they were close to the blood vessels and had a more oblique orientation. The analysis of IR% in the different areas showed that the SAT and DAT have comparable overall contents of LVs, albeit with significantly different diameters—smaller in the SAT (11.6 ± 7.7 µm) than in the DAT (22.5 ± 12.77 µm).

The close relationship of the LVs with the SF has many clinical implications, especially if we consider that the thin lymph vessels need the support of the extracellular matrix to maintain their patency. It is well known that the lymphatic cells are closely connected to the surrounding tissues by fine strands of reticular fibers and elastic fibers [29,30], called anchoring filaments. When the interstitial pressure is low, the anchoring filaments are in a relaxed conformation, resulting in the closure of oak-leaf-shaped overlapping flaps. Consequently, it easy to suppose that any alteration of the SF can affect the anchoring filaments of the LVs, reducing their ability to drain the lymph. Additionally, in the SAT, a close relationship between LVs and the fibrous septa was highlighted, suggesting that the elasticity and organization of the fibrous matrix can also affect lymphatic transport from the dermal plexus to the deeper, larger lymphatic vessels, improving or worsening it [9]. Avraham et al. [31] showed that the inhibition of fibrosis by topical dressings can accelerate the lymphatic regeneration of normal capillary lymph vessels, so an altered structure of the SF and subcutaneous layers could probably compromise the lymphatic drainage. In the same way, damage to the superficial fascia during classic abdominoplasty procedures can lead to the interruption of lymphatic vessels of a certain caliber, causing a high incidence of seroma and postoperative complications. It has already been clinically demonstrated that the classic plane of dissection—on the top of the deep fascia—should be avoided in the lower abdomen to minimize wound-healing complications and seroma [32]. Indeed, this work demonstrates that the superficial fascia plane contains a lymphatic plexus, explaining how its preservation can reduce complications and lead to greater surgical success.

Bassalobre et al. [33] showed that significant changes in the lymphatic drainage pathways occurred in the infraumbilical region after abdominoplasty, with the axillary drainage path predominating after the surgery, in contrast to the inguinal path observed in the preoperative period.

Furthermore, our new results on the distribution of LVs may improve the comprehension of clinical manifestations of impaired lymphatic transport. Several authors have already revisited the anatomy of the lymphatic system, reliably identifying the lymphatic collectors and venules [34,35,36], but we think that the knowledge of the strong relationships between fibrous elements of the subcutaneous tissues [9,37] and LVs could better improve the clinical and ultrasound evaluation of lymphedema and, consequently, improve targeted therapies, as well as suggesting modifications of manual lymphatic drainage techniques in postoperative subjects. It is well known that lymphedema can induce complications such as inflammation [38], fat tissue hypertrophy, and fibrosis [4,10], and the subcutaneous alterations [39] can alter the environment around the LVs, increasing their collapsibility. In the near future, in the evaluation of lymphedema, it will be important to also consider the thickness, texture, and stiffness of the SF, SAT, and DAT, because they may directly influence the responses to the various therapeutic interventions. It is evident that lymphatic transport is not a mere passive and automatic process [40,41] but represents a highly sophisticated system in which endothelial and muscle cells, along with the collagen and elastic fibers around them, ensure proper lymphatic drainage and propulsion [9,42,43].

## 5. Conclusions

Immunohistological analysis of lymphatic vessels has led to increased knowledge of their anatomy in the subcutis. The vessels were described considering their presence with respect to the superficial fascia and to the subdivision of the subcutaneous tissue into layers. The demonstration of different gauges of vessels led us to consider them as playing different functional roles in the collection and transport of the interstitial fluid. The results suggest new perspectives in the diagnosis and surgery of clinical manifestations caused by altered lymphatic transport. New knowledge of the lymphatic vessels will enable us to reduce post-surgical symptoms or problems and improve the outcomes of treatments.
